# High-efficiency sparse convolution operator for event-based cameras

**DOI:** 10.3389/fnbot.2025.1537673

**Published:** 2025-03-12

**Authors:** Sen Zhang, Fusheng Zha, Xiangji Wang, Mantian Li, Wei Guo, Pengfei Wang, Xiaolin Li, Lining Sun

**Affiliations:** ^1^State Key Laboratory of Robotics and System, Harbin Institute of Technology, Harbin, China; ^2^Lanzhou University of Technology, Lanzhou, China; ^3^Institute of Intelligent Manufacturing Technology, Shenzhen Polytechnic University, Shenzhen, China

**Keywords:** event-based camera, sparse convolution, convolution operator, high-efficiency, low-latency

## Abstract

Event-based cameras are bio-inspired vision sensors that mimic the sparse and asynchronous activation of the animal retina, offering advantages such as low latency and low computational load in various robotic applications. However, despite their inherent sparsity, most existing visual processing algorithms are optimized for conventional standard cameras and dense images captured from them, resulting in computational redundancy and high latency when applied to event-based cameras. To address this gap, we propose a sparse convolution operator tailored for event-based cameras. By selectively skipping invalid sub-convolutions and efficiently reorganizing valid computations, our operator reduces computational workload by nearly 90% and achieves almost 2× acceleration in processing speed, while maintaining the same accuracy as dense convolution operators. This innovation unlocks the potential of event-based cameras in applications such as autonomous navigation, real-time object tracking, and industrial inspection, enabling low-latency and high-efficiency perception in resource-constrained robotic systems.

## 1 Introduction

Low-computation and low-latency visual perception are crucial for robotic systems. Compared to the highly efficient visual processing capabilities of advanced biological systems such as humans, robotic vision often requires significantly greater computational resources (Wu et al., 2022; Meng et al., 2025). This inefficiency imposes substantial constraints on a wide range of robotic applications. A notable example can be seen in small micro aerial vehicles (MAVs). To maximize flight endurance, MAVs are typically designed to be lightweight, which restricts them to carrying low-power embedded computing devices. As a result, their visual processing capabilities are often limited to basic visual functions or extremely slow in high-level 3D vision (Guo et al., 2024; Cheng et al., 2024), frequently making timely and effective obstacle avoidance difficult. The challenge becomes even more pronounced in autonomous driving. Ensuring safety and robustness demands the simultaneous execution of multiple perception sub-tasks (Qian et al., 2022; Wang et al., 2019; Jiang et al., 2019). Moreover, integrating information across spatial, color, temporal, and multi-camera dimensions significantly increases the computational burden, making real-time perception increasingly difficult. Consequently, autonomous driving systems often struggle to meet stringent latency requirements. Their typical perception update rates reach only around 30 Hz (Yu et al., 2023), which falls far short of the ideal requirements.

**Figure 1:**
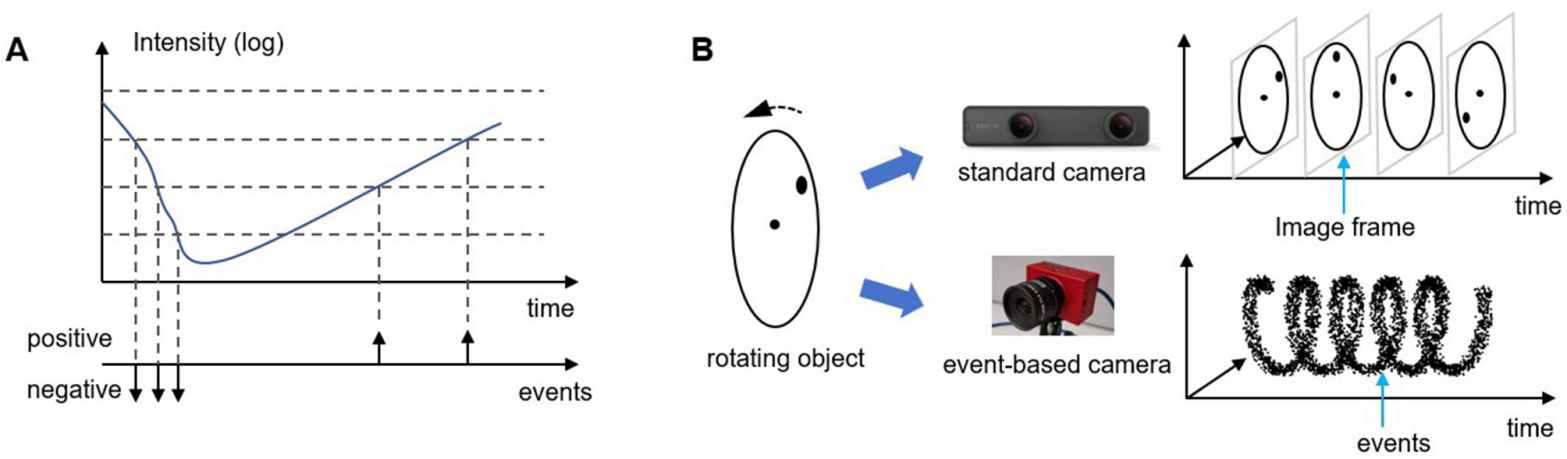
Working principle of event-based cameras. **(A)** demonstrates working principle of a single pixel, while **(B)** shows the comparison of imaging results between event-based cameras and conventional standard cameras.

**Figure 2:**
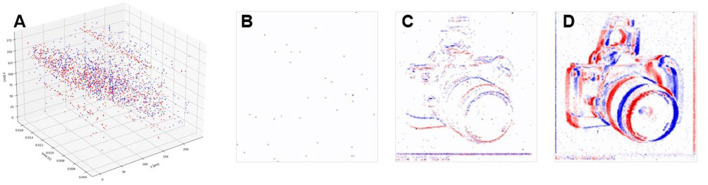
Spatial sparsity of event-based cameras, **(A)** demonstrates the events in spatial-temporal coordinate. **(B–D)** shows the 2D histogram image by accumulating events in time windows of 1 ms, 10 ms, and 100 ms. They shows the great sparsity comparing with dense image captured by conventional standard cameras.

An event-based camera is a bio-inspired vision sensor designed based on the working principles of the animal retina. Drawing inspiration from the transient visual pathway, it replicates the sparse activation and asynchronous transmission of retinal ganglion cells. Unlike conventional cameras that capture visual information by recording entire image frames, event-based cameras encode visual data through discrete “event”, as illustrated in Figure 1. This approach offers a finer and more dynamic representation of visual information, akin to how visual neurons in the animal nervous system respond selectively to changes in their environment. To achieve this, the pixels of an event-based camera remain continuously exposed, much like the human eye's constant reception of light. When the perceived light intensity fluctuates beyond a certain threshold, the corresponding pixel generates a signal. Depending on whether the intensity increases or decreases, this signal can be positive or negative. These signals, known as “event”, serve as the fundamental units of visual information in an event-based camera. An “event” captures and encodes significant changes in light intensity from the environment, selectively highlighting the most essential visual information. This biologically inspired strategy results in a highly sparse output, with visual information at any moment far less than that of conventional cameras (Rebecq et al., 2019; Miao et al., 2019). As shown in Figure 2, sparsity often reaches 99%, enabling efficient processing by reducing computational complexity and inference time.

However, despite event-based cameras have the characters of low latency and high sparsity, vision processing algorithms that fully leverage this sparsity are still relatively rare. Current robotic vision algorithms are primarily designed for conventional dense vision systems. Since event-based cameras operate on fundamentally different principles than standard cameras, applying traditional vision algorithms to event-based camera data often disregards their inherent sparsity. This leads to high computational complexity and increased latency, resulting in inefficient use of computational resources and longer inference times, ultimately hindering the potential benefits of sparse, low-latency processing. Therefore, a critical challenge lies in how to effectively utilize the sparse nature of event-based camera data and design sparse processing algorithms specifically tailored for event-based data.

Some research efforts have focused on leveraging the sparsity of event-based cameras and reducing redundant computations. One approach processes events sequentially in real-time (Brosch et al., 2015), eliminating zero-padding and improving efficiency, but requiring extensive manual design, limiting generalization to complex tasks. CNNs remain dominant in visual perception, with studies showing improved accuracy for event-based data. To optimize convolutions, some methods use hash tables (Messikommer et al., 2020) to manage sparse computations efficiently. However, hash lookups disrupt computational continuity (Sorin et al., 2022), leading to increased inference latency despite reduced computation. Similarly, Graph Convolutional Networks (GCNs) (Schaefer et al., 2022) reduce computational load but also suffer from discontinuous processing, preventing latency improvements. Spiking Neural Networks (SNNs) (Cordone et al., 2021; Orchard et al., 2015b; Bing et al., 2018; Jiang et al., 2017), inspired by biological neurons, align well with event-based data due to sparse activation and asynchronous updates. However, they struggle with accuracy compared to CNNs and often require specialized hardware, limiting their practical use. Despite progress, no existing method fully resolves the balance among computational efficiency, low latency, and high accuracy in event-based vision.

To achieve the goal of low-latency visual perception with minimal computational resources while maintaining high accuracy, this paper proposes a sparse convolution operator specifically designed for event-based cameras. By eliminating redundant sub-convolutions, the computational load is significantly reduced. Meanwhile, the valid sub-convolutions are efficiently reorganized into matrix multiplication operations, greatly enhancing the computation speed while preserving the same level of accuracy as conventional convolution operators.

The main contributions of this paper are as follows: 1. Sparse Convolution Operator: We propose a novel sparse convolution operator that significantly reduces the computational load and accelerates inference for sparse input data. To the best of our knowledge, this is the first sparse convolution operator that surpasses the inference speed of conventional convolution operators when processing event-based camera data. 2. Efficient Sub-Convolution Detection: We introduce an efficient method for detecting valid sub-convolutions based on the location information of active pixels, enabling the computation of sub-convolution indices without the need for exhaustive traversal. 3. Sparse im2col: We present a sparse im2col technique that reorganizes sparse convolution input data into a dense matrix format, allowing for efficient matrix multiplication, protecting the computational continuity and further accelerating the sparse convolution operation.

## 2 Related works

### 2.1 Sparse convolution

Image-based visual data often encounters sparse input situations, such as handwritten digits, 3D point clouds, and 3D voxel data. These types of data contain large areas of empty space, with meaningful information concentrated in only a small portion of the region. When using conventional convolutional neural networks to process this data, a large amount of empty, invalid computations are generated. Therefore, a class of methods attempts to modify the convolution operator by discarding invalid operations to take advantage of this data sparsity, aiming to reduce computation and accelerate processing. These methods are called sparse convolutions. The most classic sparse convolution approach was first proposed by Facebook (Graham and Van der Maaten, 2017; Graham et al., 2018). The authors, when solving the handwritten digit recognition problem, noticed the sparsity of the data and used hash tables to record the elements to be computed and their computational relationships, reflecting the sparsity. Since the table only records valid computations and excludes invalid operations in the empty regions, this method successfully reduces the computational load. This approach was then applied to the processing of 3D point clouds and 3D voxels (Yan et al., 2018). In practical scenarios, 3D point cloud information is generally highly sparse, with large areas of empty space. By extending the above method to three dimensions, sparse convolution has been successfully applied in the field of autonomous driving for 3D point cloud recognition, greatly improving the efficiency of 3D convolution operations for point clouds and voxels. It is worth noting that the creation of the hash table itself also consumes computational resources, and the advantages of this method are only evident when the input data is sufficiently large. For 3D point clouds, the computational delay caused by the dimensional explosion of 3D convolution is very high, highlighting the benefits of sparsity. However, for ordinary 2D images, the creation cost of the hash table is generally non-negligible. Therefore, for event-based cameras, sparse convolution based on hash tables is difficult to fully realize its potential in terms of computational delay. In addition to the hash table-based sparse convolution approach, there are other sparse convolution applications. For example, Parger et al. (2022b,a) noticed that similar content appears frequently between consecutive video frames, so the difference between two frames is used as the input data for each inference. This difference often exhibits spatial proximity and is likely to satisfy computational continuity, where sparse convolution can achieve good results with a simple approach. However, the effective data generated by event-based cameras is more unevenly distributed, making it less suitable for simple sparse convolution methods.

### 2.2 Data processing for event-based cameras

Due to the significant differences in the working principles and information representation between event-based cameras and standard cameras, corresponding visual perception algorithms also exhibit significant differences. Generally speaking, event-based camera data processing algorithms can be divided into two categories: one is event-based processing, and the other is to aggregate events into groups for processing (Gallego et al., 2020). In general, event-based processing better leverages the high event resolution and low latency advantages of event-based cameras, as there is little waiting time between event generation and processing. One typical application of this approach is in SLAM systems. By utilizing methods like Kalman filtering or particle filtering, robot pose tracking can be efficiently and quickly performed from event data. Event-based processing can also be applied to other vision tasks, such as feature extraction (Brosch et al., 2015) and image reconstruction (Munda et al., 2018). These tasks combine past event information with current data to accomplish high-level visual tasks, which to some extent align with the asynchronous nature of event-based cameras. However, a drawback of event-based processing is that it cannot provide enough effective information at once and is susceptible to noise interference, often limiting its application range and performance, particularly in high-level semantic reasoning. Aggregating events into groups allows for the simultaneous consideration of more information, making the information extraction and reasoning process more convenient. The methods of event aggregation and representation are diverse, mainly including event frames (Liu and Delbruck, 2018), Time Surface, Voxel Grid, 3D Point Sets, and others. The event frame representation allows for the reuse of conventional image processing methods, such as convolutional neural networks (CNNs), to process event-based camera data, and such methods have been proven to be highly effective in various tasks. Time Surface is a representation sensitive to motion direction and scene edges, making it particularly effective in applications such as optical flow estimation (Benosman et al., 2013). Many studies now use this approach as the foundation for dynamic feature extraction, feeding it into CNNs and other neural networks for perceptual reasoning, achieving promising results (Zhu et al., 2018). Voxel Grid and 3D Point Set representations extend the information into the spatiotemporal domain. On one hand, they retain more information; on the other hand, they demand higher computational resources. These representations can generally be input into various ANN models (e.g., CNNs) for better processing results. From this, it can be seen that convolutional neural networks are foundational modules in event-based camera data processing methods, and convolution operators play an important role in event-based camera perception and reasoning. Moreover, there are still relatively few approaches that focus on the sparsity of event data in convolutional inference.

## 3 Methods

This paper is primarily inspired by the GEMM (General Matrix Multiply) method in traditional dense convolutions. By transforming the sparse convolution for processing event-based camera data into dense matrix multiplication, it reduces the computational load while preserving the continuity of the computations, thus achieving efficient sparse convolution operations. The following section provides a detailed explanation of the specific approach.

### 3.1 Converting events to tensor

The main difference in working principles between event-based cameras and standard cameras lies in the fact that in an event-based camera, each pixel unit operates independently and asynchronously. The data generation process of each pixel unit is not controlled by a unified clock cycle, but rather by the changes in the ambient light information. The basic perceptual output of each pixel unit is called an event. An event is triggered when the logarithmic intensity of the light stimulus received by a pixel unit exceeds a preset threshold *c* compared to the previous moment, that is:


(1) $$\begin{array}{l} |log\left(I\right)-log\left(I_{last}\right)|>c \end{array}$$

It will then generate and output *event*_*i*_, which contains the pixel coordinates (*x*_*i*_, *y*_*i*_) of the pixel, the time *t*_*i*_ when the event occurred, and the polarity *p*_*i*_, which indicates whether the change in light intensity exceeded the threshold in an upward or downward direction. That is:


(2) $$\begin{array}{l} event_{i}=\left(x_{i},y_{i},t_{i},p_{i}\right) \end{array}$$

As the ambient light information changes, the event-based camera can output a continuous asynchronous event stream over time. A single event contains very little information, making it difficult to achieve the desired results through processing individual events. However, by recording all events that occur within a small time window Δ*t*, a set of events, *S*_Δ*t*_, can be obtained. This event set *S*_Δ*t*_ carries more information and provides a more meaningful representation for further processing.


(3) $$\begin{array}{l} S_{\text{Δ}t}=\left\{\left(x_{i},y_{i},t_{i},p_{i}\right)|t_{i}\in \text{Δ}t\right\} \end{array}$$

When the ambient light source remains constant, the changes in brightness within an image are typically caused by the movement of objects. Specifically, movement at the edges of objects tends to result in more significant changes in intensity. Therefore, the event set *S* generally contains information about object edges, which is crucial for visual tasks such as object detection. By using a sliding window, the contents of *S* can be continuously updated to acquire new perceptual data. Additionally, by controlling Δ*t*, one can adjust the temporal receptive field and the amount of spatiotemporal information processed in each instance.

Modern AI algorithms typically use tensors as the fundamental data structure. Considering the scalability and compatibility of the proposed convolution operator, event-based camera data *S* is expressed here in the form of an image tensor, similar to traditional images. Based on the definitions above, each *event*_*i*_ contains information in four dimensions. First, for the time *t*-dimension, when Δ*t* is sufficiently small, it can be approximated that the events in *S* occur simultaneously. In this case, the time-axis information of visual events within the Δ*t* time window is compressed. For the polarity dimension, the polarity *p* of an event has only two possible values, and events with the same polarity *p* are spatially and logically closely related. Therefore, events with different polarities *p* can be treated as information in different channels of the image, i.e., using polarity as the channel dimension. Finally, for the width and height dimensions, the *x* and *y* coordinates of *event*_*i*_ represent the pixel positions where the event occurs, which is consistent with traditional images. Thus, an event *event*_*i*_ can be represented as a multi-dimensional vector *T*_*i*_, similar to a pixel in a traditional image.


(4) $$\begin{array}{l} Ti=\left[N,p_{i},h_{i},w_{i}\right] \end{array}$$

In this representation, *p*_*i*_, *h*_*i*_, and *w*_*i*_ belong to event_*i*_, where *N* represents the batch dimension, *p*_*i*_ is the channel dimension, and *h*_*i*_ and *w*_*i*_ represent the height and width dimensions, respectively. The tensor *T*_*i*_ obtained from all events that occurred within the time window Δ*t* can be stacked to form the real-valued part of the image tensor. However, there are many pixel positions within the time window Δ*t* where no visual events occurred. For these positions where no event has taken place, zeros are used to fill the corresponding locations. In this way, we obtain a data representation in the form of a tensor *ET*, which is compatible with modern computer vision techniques. This tensor *ET* provides a structured representation of event-based camera data that can be processed using standard AI algorithms.


(5) $$\begin{array}{l} ET=0+\sum Ti \end{array}$$

The *x*_*i*_ and *y*_*i*_ in *event*_*i*_ record the positions of valid pixels in *ET*. We will denote the set of all valid pixel positions in *ET* as *Valid*_*pix*. These are the positions where events have occurred and contain meaningful visual information, distinguishing them from the positions that remain empty (where no event has been triggered).


(6) $$\begin{array}{l} Valid\text{\_}pix=\left\{\left(x_{i},y_{i}\right)|x_{i},y_{i}\in event_{i},event_{i}\in S_{\text{Δ}t}\right\} \end{array}$$

Since the *ET* tensor contains a large number of zero elements, performing operations such as convolution directly would result in a significant amount of meaningless redundant computation. The design of an efficient sparse convolution algorithm that leverages data sparsity will be discussed in detail below.

### 3.2 Valid sub-convolution detection

Convolutional neural networks (CNNs) are an important method of information processing in robotic vision, with the convolution operator being the core of the network. The convolution operation with an image is performed by convolving the kernel with data from a specific window in the input image, which is then traversed across the image using a sliding window. Due to the sparsity of the input image data, the sub-convolution operation at specific windows may contain a certain number of zero computations. When a sub-convolution still contains valid computations, it is called an valid sub-convolution. When a sub-convolution contains only invalid zero computations, it is called an invalid sub-convolution. We denote the index set of valid sub-convolution as *Valid*_*subconv*. valid sub-convolutions are operations that have an impact on the inference results and cannot be ignored. The challenge in achieving efficient sparse convolution lies in how to organize and transform valid sub-convolutions into efficient computations. Before this, it is necessary to first detect the specific locations of the valid sub-convolutions. Thanks to the efficiency of the event-based camera's raw data representation, we can easily obtain the positions of valid pixels in the *ET* tensor, denoted as *Valid*_*pix*, as described in Equation 6. There is a strong correspondence between the positions of valid pixels and the positions of valid sub-convolutions. A window containing an valid sub-convolution must necessarily include valid pixels, and each valid pixel corresponds to a specific valid sub-convolution. Therefore, the locations of the valid sub-convolutions can be directly inferred from *Valid*_*pix*. Specifically, except at the image edges, each valid pixel corresponds to K sub-convolution operations, where K is the size of the sub-convolution window. By matching this pixel with the elements at different positions in the convolution kernel, we can determine the positions of the K sub-convolutions. At the image edges, the number of sub-convolution operations corresponding to each pixel will be fewer than K, and additional checks are required to verify whether the positions of the sub-convolutions are correct and valid. Since the positions of all valid pixels are recorded in *Valid*_*pix*, we can traverse *Valid*_*pix* and take the union of all the positions of the valid sub-convolutions to obtain the complete set of valid sub-convolution positions. This approach greatly improves time efficiency compared to the method of detecting valid sub-convolutions by directly traversing according to their definition.

### 3.3 Sparse Im2col

**Figure 3:**
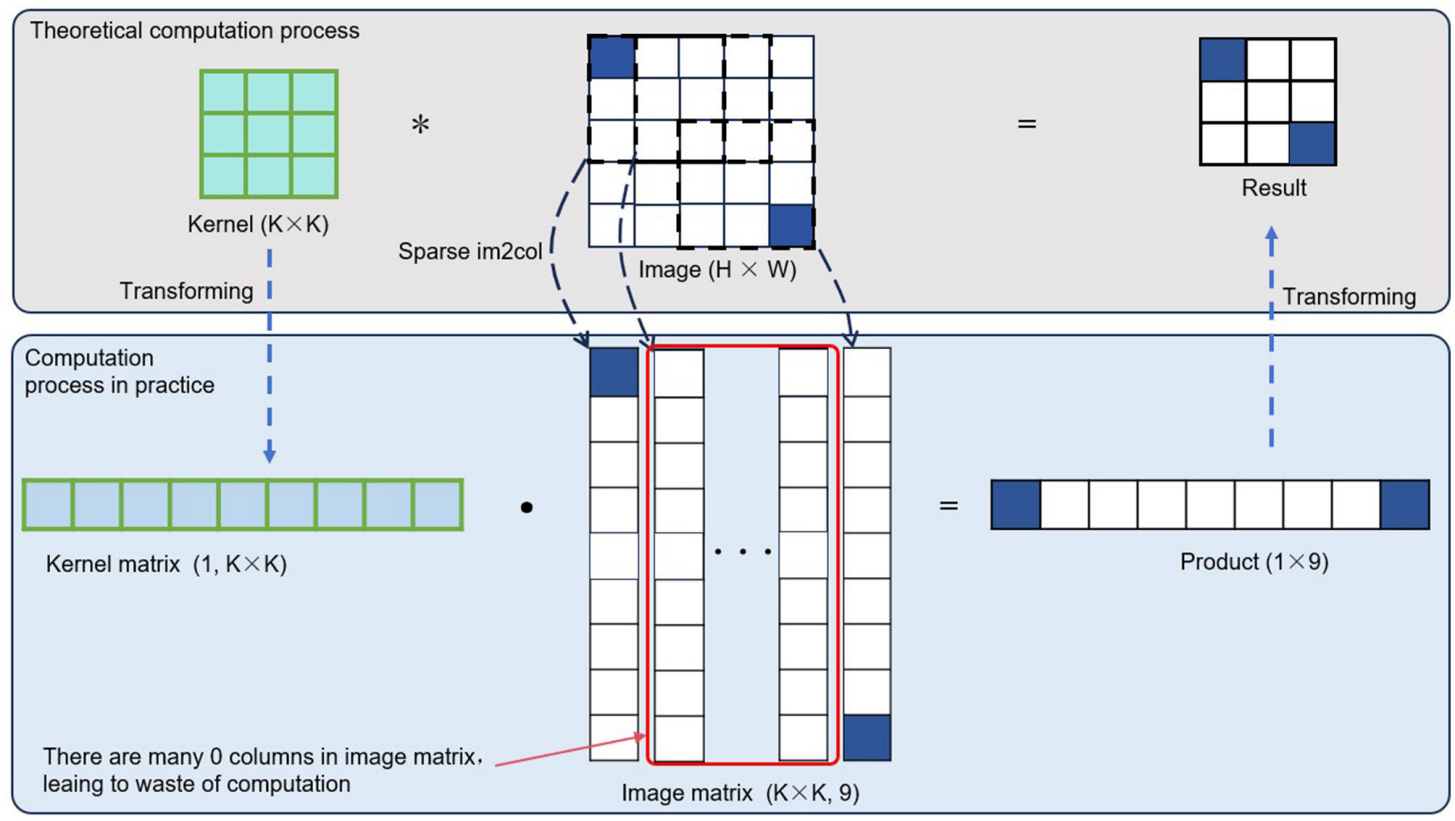
Conventional dense convolution is usually transformed into general matrix multiplication (GEMM) when executed on actual chips. This transformation helps maintain computational continuity and significantly improves processing speed compared to a naive implementation that strictly follows the definition. It can also be observed that when the input is a sparse tensor, a significant number of redundant computations occur in the GEMM operation. If these redundant computations are eliminated, the overall computation speed can be further improved. The asterisk (*) denotes the “convolution operator,” that is, the convolution in convolutional neural networks.

The convolution operation with an image is essentially a series of logically parallel multiplication and addition operations, forming a very regular computational structure. In practice, however, this often transforms into a more computer-friendly format for parallel computation, specifically general matrix multiplication (GEMM). By converting the convolution operation into GEMM form, the computation speed can be significantly accelerated. To facilitate the introduction of the computational organization involved in this invention, let's first explain the general principle of conventional dense convolution operations. In typical image convolution, the convolution kernel is unfolded into a row vector, and the convolution kernel vectors of different channels form a convolution kernel matrix. The image data in the convolution operation is unfolded into column vectors, and the combination of data from different channels and batches forms an image matrix. By multiplying the resulting convolution kernel matrix with the image matrix, a single general matrix multiplication operation is performed, yielding the result of the image convolution operation (though the element ordering may differ slightly). The overall computation process is shown in Figure 3. Since typical dense image convolution operations do not account for invalid calculations, i.e., multiplication and addition operations involving zero elements, processing event-based camera data in this way would waste considerable computational resources, slowing down the operation time.

This paper introduces an innovation in the image matrix processing step. By removing invalid computation data, the remaining valid operations are transformed into general matrix multiplication (GEMM) operations to achieve efficient computation. The specific approach is as follows: First, for each valid sub-convolution, following the general image convolution method, the image data of one channel in the convolution window is unfolded into a column vector in a row-major order. Then, the column vectors obtained from different channels are concatenated by columns to form the column vector corresponding to the current valid sub-convolution. Next, all the valid sub-convolution operations are traversed in the order defined in *Valid*_*subconv*, and the above steps are repeated to convert all the image data required for valid operations into several column vectors. Finally, all the column vectors corresponding to the valid sub-convolutions are placed into adjacent memory spaces and concatenated by rows to obtain an image matrix. This matrix is then used for general matrix multiplication. In typical dense image convolution operations, the process of converting the image into a matrix for GEMM is called im2col. Therefore, in this paper, the process described above is referred to as sparse im2col. As for processing the convolution kernel matrix, it remains consistent with the general image convolution algorithm. Compared to the im2col in typical image convolution, sparse im2col does not require traversing the entire input tensor directly. It only needs to traverse the smaller, valid convolution portion, bringing two main benefits. On one hand, the computational load for the conversion process is greatly reduced, enabling faster conversion from convolution to GEMM. On the other hand, the resulting matrix is smaller, reducing the computer's memory consumption, and a smaller matrix also means a significant reduction in the computational load for subsequent matrix multiplication operations.

**Figure 4:**
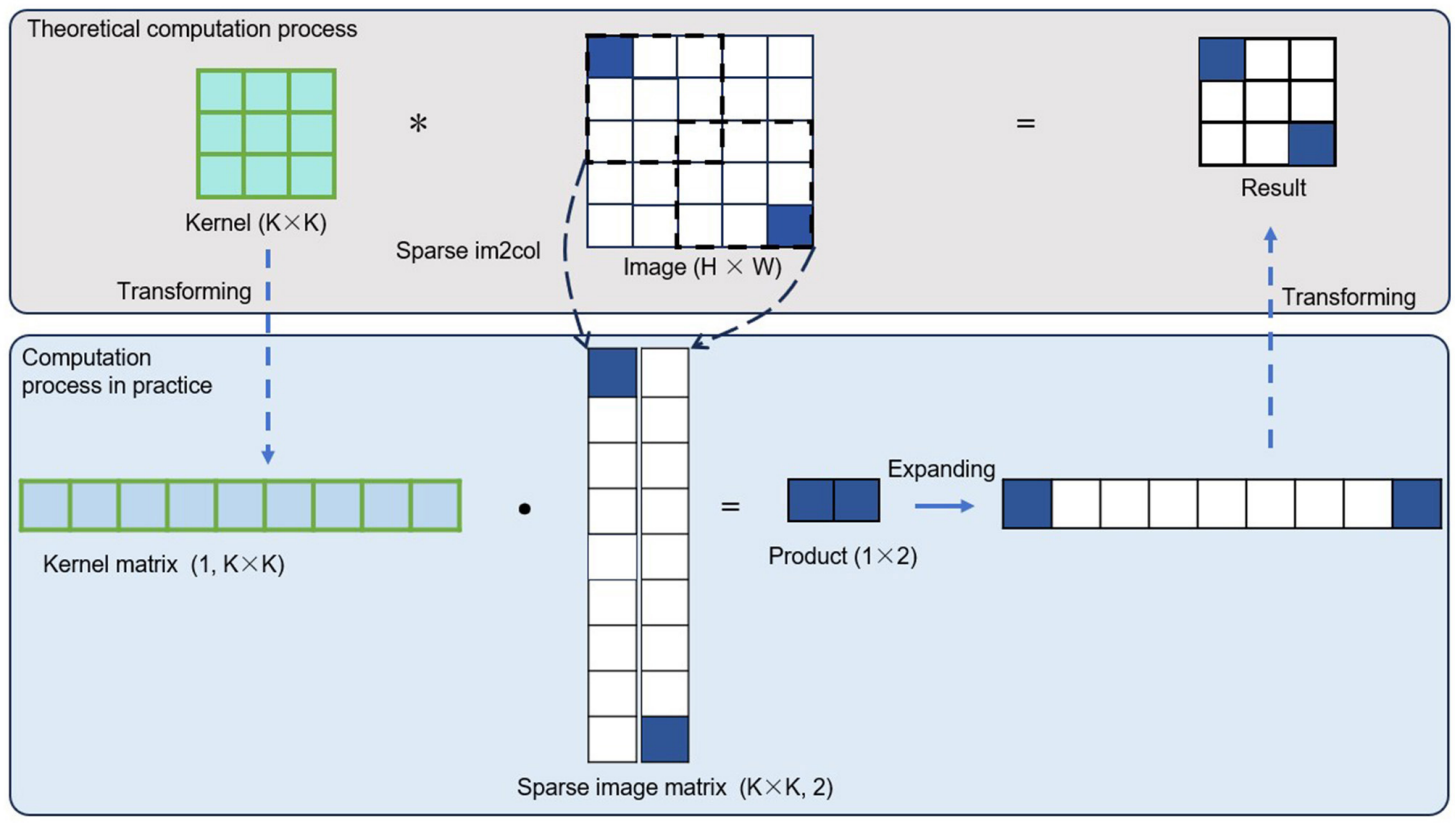
We first eliminate redundant computations in the GEMM to reduce the overall computational cost. Then, we organize the remaining valid operations into a GEMM format again to preserve computational continuity. The combination of eliminating redundant computations and preserving computational continuity is the key distinction that sets our method apart from traditional dense convolutions and other sparse convolution approaches. The asterisk (*) denotes the “convolution operator,” that is, the convolution in convolutional neural networks.

Figure 4 provides a visual representation of the process described above. For simplicity, only the single-channel convolution is shown, though the actual process is more complex. In the case of typical dense image convolution operations, the matrix size corresponding to the convolution kernel is [*channel*_*out, kernel*_*size* × *kernel*_*size* × *channel*_*in*], and the matrix size corresponding to the image data is [*kernel*_*size* × *kernel*_*size* × *channel*_*in, Valid*_*subconv*]. In general image convolution operations, the matrix size corresponding to the image data is [*kernel*_*size* × *kernel*_*size* × *channel*_*in, N*×*h*_*out* × *w*_*out*]. Therefore, the computational cost ratio of the proposed oprator to that of standard image convolution can be calculated as η = *Valid*_*subconv*/(*N* × *h*_*out* × *w*_*out*). That is the key reason behind the high efficiency of the method. This paper examines the computational sparsity at the sub-convolution level, retains only the valid sub-convolutions, and organizes the remaining valid sub-convolutions into general matrix multiplication operations. This approach not only greatly reduces the computational load but also ensures hardware friendliness in computation.

### 3.4 Converting the result as regular convolution

The general image convolution operation completed by general matrix multiplication yields a matrix product result *Pd*, which is equivalent to the theoretical convolution result. However, the general matrix multiplication operation completed in section above, which focuses only on the non-zero elements, results in a product *Ps* that differs from the typical image convolution operation. It is a subset of the full convolution result that contains only the valid information. To be compatible and adaptable with other computational components in modern neural networks, such as max pooling and batch normalization, it is necessary to further transform *Ps* into a form identical to that of a typical image convolution. The specific approach is as follows: first, fill the corresponding positions in the resulting product matrix *Ps* with zero elements, expanding it to the same size and shape as the matrix multiplication result *Pd* in typical image convolution. This can be achieved by utilizing *Valid*_*subconv* again. Based on the above description, the following relationship exists between *Ps* and *Pd*:


(7) $$\begin{array}{l} P_{s}=\left\{p_{i}\in P_{d}|i\in Valid\text{\_}subconv\right\} \end{array}$$

That is, the result obtained by combining the columns of *Pd* at the index positions of the elements in *Valid*_*subconv* is *Ps*. This can be easily explained, as in matrix multiplication, the columns of the product correspond one-to-one with the columns of the operand matrix. Therefore, by using the above relationship in reverse, we can remap the elements in *Ps* to new positions based on *Valid*_*subconv*, and fill the complement positions of *Valid*_*subconv* with zero to obtain the desired result, denoted as *Pds*. Next, *Pds* needs to be further transformed into the format of a typical image convolution. The result *Pds*, obtained from general matrix multiplication, has a data layout format of [C × N × H × W], whereas modern neural networks typically use the [N × C × H × W] format. Thus, by swapping the axes, the final required image convolution result can be obtained.

## 4 Results

To verify the correctness and effectiveness of the proposed method, we designed a neural network with a single convolution layer and evaluated the proposed sparse convolution operator from three dimensions: computational accuracy, computational complexity, and actual inference time. For the input data, we selected the N-Caltech101 dataset (Orchard et al., 2015a), which is derived from real event-based camera recordings. This dataset captures images from the Caltech101 dataset using an event-based camera, generating corresponding event data. It contains 8,246 samples across 101 categories, with each sample comprising 300 ms of event-based camera data. The image resolution is 180 × 240. The experimental platform utilized an Intel i7-12700KF CPU as the computing unit, with 16GB of memory and running Ubuntu 20 as the operating system. The code was compiled using gcc with the -O3 optimization option enabled.

The primary factor influencing the inference performance of a sparse convolution operator is the sparsity level of the input data. In theory, the higher the sparsity, the more evident the advantages of the sparse convolution algorithm. In the extreme case where the sparsity level is zero, the algorithm degenerates into a dense convolution. To investigate the performance of the proposed sparse convolution operator under varying sparsity levels, we generated input data with different sparsity by extracting event data from time windows of different lengths within the samples.

To more clearly demonstrate the advantages of the proposed sparse convolution operator, we conducted comparative experiments with both dense convolution and the classical SCN sparse convolution method. For the dense convolution implementation, we selected the standard dense convolution operator from Intel's MKL library, which offers the highest inference efficiency for convolutional neural networks on Intel CPU products. This is due to extensive engineering optimizations specifically tailored to the characteristics of Intel CPUs.

In the N-Caltech101 dataset, each 300 ms sample is recorded by moving the camera in three different directions relative to a static image, with each movement lasting 100 ms. Therefore, the first 100 ms of data can be considered representative of the entire sample. To generate data with varying sparsity levels, we extracted time windows of different sizes from this 100 ms segment, starting from 0 ms, with a time step of 1 ms, resulting in 100 sets of event-based camera data with different densities. The raw event data is then converted into a tensor following the previously described method, yielding an input tensor with the shape [8, 2, 180, 240]. When the time window is set to 100 ms, the maximum density of the input tensor reaches approximately 3%.

First, we validate the computational accuracy. Theoretically, the proposed convolution operator only eliminates redundant multiplications and additions involving zeros, so its results should be identical to those of standard dense convolution. We randomly initialize the weights for the dense convolution and assign the same weights to the proposed sparse convolution operator. Using PyTorch's allclose function, we verify whether the results are identical. The relative and absolute tolerance values are set to 10^−3^ and 10^−5^, respectively. The statistical results show that the outputs of both methods fall within the tolerance range, consistent with theoretical expectations, confirming the correctness and reliability of the proposed sparse convolution operator.

**Figure 5:**
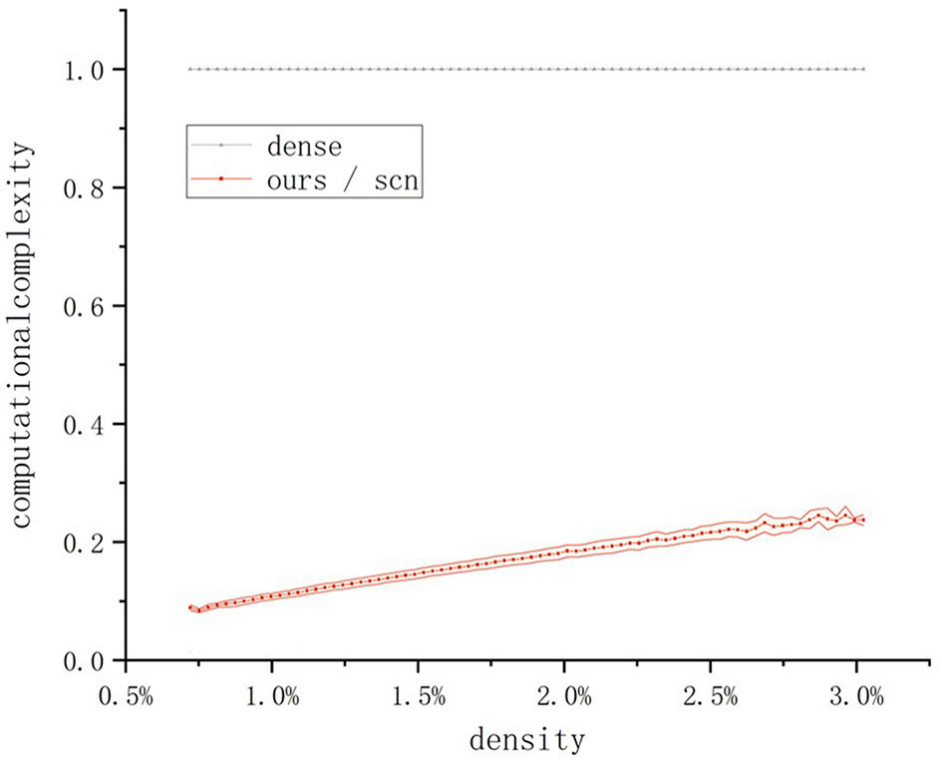
Comparison of the computational complexity of different convolution operators. The computational complexity of the method in this paper is the same as that of the SCN method. The line represents the average complexity, and the shaded envelope represents the standard deviation of the complexity.

Next, we conduct a comparative experiment on computational cost. Since the primary operations in the convolution process are multiplications and additions, we use the number of multiply-add operations as the basis for comparison. To clearly illustrate the differences in computational cost between the operators, we normalize the computational cost using the dense convolution as the baseline. The results are shown in the Figure 5.

It can be observed that the proposed sparse convolution operator has the same computational cost as the classical SCN sparse convolution algorithm, both of which are significantly lower than that of dense convolution. Even when the time window is set to 100 ms, where the input tensor contains the majority of the effective information, the computational cost is only about 25% of that of dense convolution.

**Figure 6:**
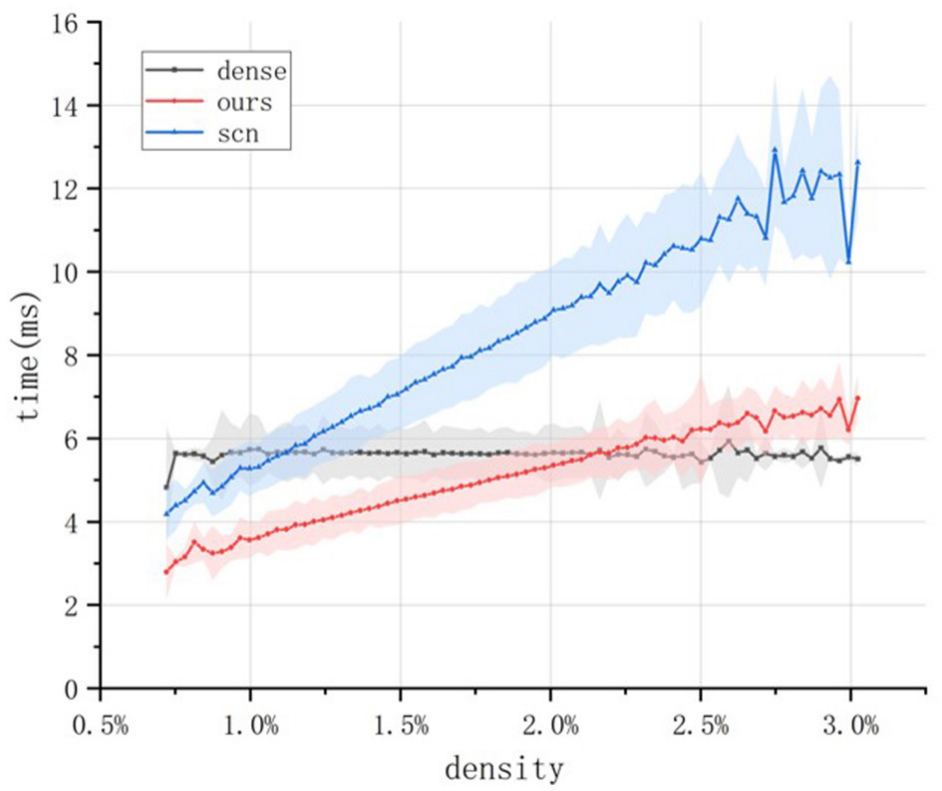
Comparison of the actual inference speed of different convolution operators. For each time window setting, the N-Caltech101 test set is traversed once to ensure multiple experiments for each sparsity level. The line represents the average inference time, and the shaded envelope represents the standard deviation of the inference time.

Next, we conducted an experiment to measure the actual inference time. The 100 input tensors mentioned above were fed into the standard dense convolution operator, the SCN sparse convolution operator, and the proposed sparse convolution operator, respectively. By evaluating the samples from the N-Caltech101 test set, we obtained the inference performance of the three operators under different input sparsity levels, as shown in Figure 6.

The experiment shows that as sparsity increases, the inference time of the SCN algorithm quickly surpasses that of dense convolution, performing better only when the data density is extremely low. However, once the density rises to around 1%–corresponding to a time window of approximately 10 ms–the inference speed of SCN falls behind dense convolution, limiting its practical applicability. In contrast, the proposed sparse convolution operator maintains competitive performance until the sparsity decreases to around 2.2%, with a corresponding time window of approximately 65 ms. Moreover, the proposed operator consistently outperforms the SCN convolution operator in terms of inference time, demonstrating superior inference efficiency.

In summary, the experimental results demonstrate that the proposed sparse convolution operator not only significantly reduces computational cost but also effectively translates this reduction into shorter inference time, while maintaining the accuracy of the inference results. This achieves efficient and accurate sparse convolution inference.

## 5 Discussion

The sparse convolution operator proposed in this paper strikes a good balance between computational complexity and actual inference time, breaking the previous situation where sparse convolution operators designed for event-based camera data achieved significant computational advantages but showed no clear benefits in practical inference. This is mainly because the sparse convolution operator proposed in this paper not only ensures sparsity but also aligns with the computational continuity characteristics of computing devices, generating only minimal additional computational overhead during the organization of operations.

Furthermore, the method proposed in this paper has an additional advantage over other sparse convolution algorithms, such as SCN: the input and output of the sparse convolution operator proposed here are consistent with conventional convolution operators, allowing for interchangeability without the need for specialized conversion between sparse and dense representations. This provides more flexibility in building neural networks.

However, the sparse convolution operator proposed in this paper also has certain drawbacks. Specifically, as the sparsity of the input data decreases, its computational efficiency advantage over conventional convolution operators gradually diminishes and eventually disappears. This is mainly because conventional convolution operators also utilize a large number of engineering optimization techniques. In contrast, the sparse convolution operator presented here is a preliminary prototype. But this also highlights the superiority of the idea of sparse convolution approach proposed in this paper. The author believes that the method proposed in this paper is not in conflict with other optimization techniques in conventional convolution operators, and that the performance advantage will become even greater once these engineering optimizations are incorporated in the future.

## 6 Conclusion

In this paper, we present an efficient sparse convolution operator tailored for event-based cameras and validate its advantages through extensive experiments. Unlike traditional methods, our approach harnesses matrix multiplication to maintain operational continuity, effectively transforming reduced computational complexity into a substantial decrease in inference time. Remarkably, our operator reduces the computational workload by nearly 90% while nearly doubling processing speed, all while preserving the accuracy of dense convolution operators.

Thus far, our research has primarily focused on optimizing the implementation of a single convolution operator. Given that our operator maintains compatibility with conventional convolution operators in terms of input/output formats and computational processes, we will next extend its application to complete convolutional neural networks, which will enhance robotic perception and responsiveness in high-speed, emergency scenarios, providing a robust safety guarantee for large-scale real-world applications.

## Data Availability

The raw data supporting the conclusions of this article will be made available by the authors, without undue reservation.
